# Critiquing the Canadian Model of Client-Centered Enablement (CMCE) for Indigenous Contexts

**DOI:** 10.1177/00084174211042960

**Published:** 2021-10-19

**Authors:** Carly Hunter, Tara Pride

**Keywords:** Critical reflexivity, Cultural safety, Decolonizing, Epistemology of practice, Indigenous Peoples, Occupational therapy models, Professionalism, Décolonisation, épistémologie de la pratique, modéles d'ergothérapie, peuples autochtones, professionnalisme, réflexivité critique, sécurité culturelle

## Abstract

**Background.** The Truth and Reconciliation Commission of Canada outlines the need for health care professionals to create more welcoming spaces for Indigenous Peoples. The scope of occupational therapy is continually expanding—yet the profession itself is grounded in and derived from a dominant Eurocentric worldview, and practice is designed to serve a homogenous Western populace. **Purpose.** To critically examine the Canadian Model of Client-Centered Enablement (CMCE) for its value within Indigenous contexts. **Key Issues.** The CMCE is positioned as a client-centered model, however there is a clear hierarchical client-professional relationship threaded throughout. Concepts such as enable, advocate, educate, coach, and coordinate demonstrate paternalistic authority, lacking reciprocity, knowledge-sharing, and power redistribution. **Implications.** Reimagining health care relationships as entrenched in social interconnectedness demands critical reflection and action. A model of practice that endorses social change and actively addresses colonial power inequities must root its paradigmatic foundations in postcolonial views of health care as a social relationship.

## Abrégé

**Description.** La Commission de vérité et de réconciliation du Canada souligne la nécessité pour les professionnels de la santé de créer des espaces plus accueillants pour les peuples autochtones. Le champ d’application de l’ergothérapie ne cesse de s’étendre alors que la profession elle-même est fondée sur une vision eurocentrique dominante du monde et que la pratique est conçue pour servir une population occidentale homogène. **But.** Examiner de façon critique le Modèle canadien de l’habilitation centrée sur le client (MCHCC) pour déterminer sa valeur dans les contextes autochtones. **Questions clés.** Le MCHCC se positionne comme un modèle centré sur le client, mais la relation client-professionnel est clairement hiérarchisée. Des concepts tels que engager, revendiquer, éduquer, coacher et coordonner démontrent une autorité paternaliste, manquant de réciprocité, de partage des connaissances et de redistribution du pouvoir. **Implications.** Réimaginer les relations de soins de santé comme étant ancrées dans l’interconnexion sociale exige une réflexion et une action critiques. Un modèle de pratique qui soutient le changement social et s’attaque activement aux inégalités de pouvoir coloniales doit enraciner ses fondements paradigmatiques dans les vues postcoloniales des soins de santé en tant que relation sociale.

## Introduction

Throughout the past several thousand years, Indigenous Peoples in what is now referred to as Canada have been living and practicing their own traditional, cultural, ceremonial, and health practices. Indigenous Peoples have been skillful and resourceful in ensuring that the health and prosperity of their peoples continues, despite numerous genocidal efforts by colonial settlers to eliminate Indigenous Peoples and assimilate them to Eurocentric worldviews and practices (Smith, 2018). Colonialism has been a detrimental force imposed upon Indigenous Peoples, creating mass destruction in their lives in various ways including the Residential School system, the implementation of reserves, and the 60's Scoop (Allan & Smylie, 2015). Notably, colonialism has been described as a distal determinant of health for Indigenous Peoples in Canada (Czyzewski, 2011; Reading & Wien, 2009), with purposeful, calculated assimilatory and genocidal policies and practices creating inequities between Indigenous Peoples and the remainder of the Canadian population. As a foundation of Canadian society, colonialism has also been built into dominant worldviews, perceptions, and practices, including those of occupational therapy. While the development of professional theories, models, and assessments by and for Indigenous Peoples is the longer-term ideal, in the immediate term critical analysis of widely used tools and resources through a decolonizing lens is needed. This paper interrogates the Canadian Model of Client-Centered Enablement (CMCE) for its value within Indigenous contexts.

### Indigenous Health and Social Inequities: A Product of Colonialism

The inequities experienced by Indigenous Peoples due to colonialism and assimilation practices on behalf of the Canadian government cannot be understated and include but are not limited to: lower educational attainment (Statistics Canada, 2011), poorer access to basic necessities such as secure and quality housing (National Collaborating Centre for Aboriginal Health, 2017), clean drinking water (Human Rights Watch, 2016), food security (Power, 2008), lower socioeconomic status (SES) (Adelson, 2005), and inadequate access to health care services and programs (Reading & Wien, 2009). It is important to ground these inequities in relation to the broader social and economic conditions, where Indigenous Peoples are not afforded the same resource investment (Commission on the Social Determinants of Health, 2008), and access to opportunities as the dominant settler population. The social, material, and health inequities faced by Indigenous Peoples today are a direct result—not of Indigenous cultures and practices—but of colonial events, policies, practices, and programs. The Indian Act (passed in 1876, modified numerous times thereafter; Henderson, 2006) is one prominent example of a legislative mechanism of colonialism. These colonial foundations are a tool for White supremacy; and are used as justification to enact genocide and perpetuate racism against Indigenous Peoples (Smith, 2012)—reinforcing the superiority of White people and the subordination of Indigenous Peoples in society.

One of the most notable inequities created by historical and current practices of colonialism is evident in the health care system. Across the board, Indigenous Peoples have inadequate access to health care services (National Collaborating Centre for Indigenous Health, 2019), and when accessing care, routinely experience systemic racism and colonial worldviews (Lowrie & Malone, 2020; Paradies, 2016) that result in inadequate and often harmful care (Allan & Smylie, 2015; White & Beagan, 2020). Canada boasts about and is respected for its inclusivity and multiculturalism, along with a high standard of living and universal health care, however, the health status indicators of Indigenous Peoples who have lived on this land for millennia are comparable to those of low resource countries (Gionet & Roshanafshar, 2013). These inequities in health status are directly related to imperialism, capitalism, and colonialism (Smith, 2018).

Spurred by influential documents such as the United Nations Declaration of Rights for Indigenous Peoples (endorsed last by Canada in 2010; not yet implemented with the exception of BC; Government of Canada, 2017), the Final Report for the National Inquiry into Missing and Murdered Indigenous Women and Girls (National Inquiry into Missing and Murdered Indigenous Women and Girls (NIMMIWG), 2019), and Canada's Truth and Reconciliation Commission Report (TRC, 2015) detailing the need for immediate action, the Canadian government has made many promises to address systemic issues affecting Indigenous communities such as unsafe drinking water (Gerster & Hessey, 2019). Thus far, the government has largely failed to turn these promises into reality, making it difficult for many Indigenous Peoples to trust that calls for change will be met with concrete action to reduce health inequities (Martens, 2019; Mitrou et al., 2014). In response to the TRC (2015) calls to address disparities in Indigenous health and wellbeing, there have been broad efforts to increase the representation of Indigenous health care professionals, and many health care providers are working to create more welcoming spaces for Indigenous Peoples accessing care, as well as those who wish to work in the health system to better support their own people and communities.

### Colonialism and Occupational Therapy

Occupational therapists need to do the work of deconstructing and actively resisting the ways colonialism is embedded in our institutions and practices to address the TRC Calls to Action (Canadian Association of Occupational Therapists (CAOT), 2018). Occupational therapy was first conceived during the First World War, where therapists during that time provided vocational training to wounded or disabled soldiers in order to support meaningful independence and engagement for their lives post-war (Canadian Association of Occupational Therapists, 2016a). Since then the profession has moved into a broad range of spaces and practice areas, yet despite diversifying its scope of practice, the profession itself has been and continues to be dominated by a singular Eurocentric worldview. The profession is grounded in and developed from this dominant perspective, and is noted to perpetuate colonialism, “when theories, assessments, interventions, outcome measures and models of practice that are informed by Western, culturally specific assumptions about what is valuable, desirable and good, are promoted and applied in contexts that are politically, culturally, economically and socially dissimilar” (Hammell, 2019, p. 19). When such cultural imposition goes uninterrogated or acknowledged, it renders it invisible and difficult to change.

A Eurocentric worldview and the values, ideologies, and norms that go with it are an ill-fitting imposition when working with the diverse communities that the profession is currently aiming to serve. It is important to note that the profession itself has actively participated in colonization through means such as Indian Day Hospitals (Meijer Drees, 2013). As such, previous critiques of occupational therapy theories have suggested that, as a profession, we should be skeptical about the extent to which theories claim or imply universality (Hammell, 2009). Those ostensibly universal assumptions and values may in fact be Western colonial impositions. For example, the narrow way self-care, productivity and leisure structure the profession; the use of child-development milestones based on Eurocentric expectations; the importance of time- and goal-orientation; the emphasis on Eurocentric values and experiences in assessments; the emphasis on objectifying and scoring performance in assessments; the notion that assessing occupational performance is safe and politically neutral (Gerlach, 2018); the standardizing of assessment tools on narrow segments of a population—all of these become cultural impositions that are harmful for Indigenous Peoples. The client-centered holistic view advocated by the profession remains superficial at best. In order for occupational therapy to embody its claims of client-centeredness and inclusivity, we need sustained critical reflection on the assumptions which underpin the profession, such as those embedded in dominant theories and models of practice (Hammell, 2015).

Currently, there is a demand from Indigenous occupational therapists within Canada to move occupational therapy towards engaging meaningfully in reconciliation with Indigenous Peoples, communities, and organizations (CAOT, 2018; Restall et al., 2016), along with a push from Indigenous Peoples and communities to have access to equitable health care services (Allen et al., 2020; TRC, 2015). This is evidenced in part by the creation of the Occupational Therapy and Indigenous Health Network (OTIHN) which “consists of CAOT members with an interest in building capacity, lobbying for occupational therapy services, and generating a greater discourse on occupational therapy and Indigenous Peoples health in Canada” (Canadian Association of Occupational Therapists (CAOT), 2016b, para. 1). Despite this, the profession lacks practice models and assessments derived from or grounded in, Indigenous ways of knowing and being. Widely used practice models and assessments are created by and designed for Western clients and therapists, identifying areas of concern based on Western epistemologies. There is little room for these foundational tools of occupational therapy to be adapted for effective use with Indigenous clients and communities.

It is important to acknowledge that the value of occupational therapy should be considered in each context when working with Indigenous clients and communities. Should there be a need for occupational therapists within this space—the ideal would be to co-create practice models, theories, and assessments alongside Indigenous Peoples, communities, and organizations to ensure relevancy, given the absence of frameworks that take into account Indigenous culture poses a significant barrier to adequate care (Greenwood et al., 2017, 2018). Examples of this work currently underway include the development of a cognitive assessment for Indigenous Australians and Torres Strait Islanders (Westphal, 2013), and an Australian interprofessional therapy outcome tool (Hill et al., 2020; Sheahan et al., 2019). Canada has seen the development of an assessment for mental health and addictions (the Native Wellness Assessment, https://thunderbirdpf.org/about-tpf/scope-of-work/native-wellness-assessment/) and a cognitive assessment for older Anishinaabe adults (Jacklin et al., 2020). In the absence of tools and interventions grounded in and derived from Indigenous epistemologies, however, occupational therapists are having to get creative and adapt current resources to the best of their abilities until the profession can truly make a meaningful shift toward more culturally relevant models and tools for use with Indigenous communities. It is important to note, however, that Indigenous Peoples and communities are incredibly diverse—this means that tools and interventions grounded in Indigenous epistemologies are not necessarily relevant or useful for other Indigenous communities. This requires our tools and interventions to be co-created in partnership with Indigenous Peoples and communities and be grounded in context.

Starting from principles of cultural safety (Papps & Ramsden, 1996), cultural humility and critical reflexivity (Beagan & Chacala, 2012), and decolonization (Tuck & Wayne Yang, 2012), the emphasis of this shift needs to focus on changing conventional practice within the profession, rather than interpreting Indigenous health inequities as a matter of cultural differences. Adopting a reconciliatory stance, occupational therapy needs to work with Indigenous Peoples, communities, and organizations toward recognizing and changing the colonial foundations that establish the expectation that Indigenous Peoples conform to the health care system. Without a deeper interrogation of the roots of colonialism within the profession, occupational therapists’ attempts to engage in reconciliation remain limited to superficial changes that do not fundamentally change Indigenous Peoples’ health care experiences. There are many people around the world doing the work of imagining what education and health care may look like through a decolonized lens (e.g., Gaudrey & Lorenz, 2018; George, 2019; Gibson, 2020) and the steps needed to move towards reconciliation.

### The Canadian Model of Client-Centered Enablement (CMCE)

A model that is frequently used within occupational therapy in Canada, yet has not been critically assessed or questioned in relation to use in Indigenous contexts is the CMCE (see Appendix A). The CMCE is a foundational practice model defining what occupational therapists do as health care professionals, detailed in the text *Enabling occupation II: Advancing occupational therapy vision for health, well-being, and justice through occupation* (Townsend et al., 2007). It is one of the core models taught to therapists across Canada and helps form the foundation for how therapists conceive of their work and relationship with clients. Thus, the CMCE comprises a pivotal influence on how occupational therapists may practice in Indigenous contexts. The purpose of the CMCE is to establish enablement as the core competency of occupational therapy, as well as outline what enablement means within the field and how to deploy specific skills and roles to foster enablement as the assumed primary goal of working with clients (Townsend et al., 2007). The analysis presented here aims to critically examine the CMCE for its use within an Indigenous context, along with providing some suggestions for improvement.

## Positionality

This paper emerged from a project completed by the first author for an Advanced Practice Issues course co-led by the second author. As authors, our own experiences as well as individual and social identities shape how we view the world, as well as our decisions and actions. As such, our own positionality inevitably influences our work. The first author is a white settler of Irish-Scottish descent who grew up in a largely homogenous white rural community in Southwestern Ontario. It was not until they got involved in Indigenous student community advocacy during undergraduate studies around various social justice issues such as MMIWG (NIMMIWG, 2019), environmental protection, and advocating for mandatory inclusion of Indigenous content within all university programs that they began to gain a broader perspective on how colonization shapes Canadian society. In part that meant recognizing that the issues facing Indigenous communities are not Indigenous issues but everyone's responsibility, especially settlers whose communities uphold colonial institutions. The second author is a Mi’kmaw woman who (due to colonial disruptions of family and community) grew up outside of her community and did not have the opportunity to understand and experience her Indigenous culture and ancestry. As such, the author grew up largely influenced by her maternal roots (non-Indigenous). It is through this resurgence of Indigenous identity and experiences that her work is positioned and shaped. Both authors argue that reflexivity is an essential component of the research process as well as occupational therapy practice—where reflecting on this relationship requires us to reflect on how our own assumptions and experiences affect the ways in which we frame this manuscript and conceptualize these discussions (Hsiung, 2010).

## Discussion

As a model of practice for occupational therapy, the CMCE is encoded in a schematic diagram which symbolizes the client-professional relationship, along with the ten enablement skills an occupational therapist uses to enable clients to engage in occupations (adapt, advocate, coach, collaborate, consult coordinate, design/build, educate, engage, specialize). Though the CMCE highlights concepts that would be important when working with Indigenous Peoples, such as inclusion, justice, and power inequalities, the model itself is developed within a colonial, Eurocentric context, which is the historical and present context of Canadian occupational therapy. Interrogating how these influences are embedded within the model is an important component of enacting cultural safety and critically examining the ways occupational therapy as a profession may cause harm to Indigenous Peoples. Applying Two-Eyed Seeing (Bartlett et al., 2012), we aim to identify the strengths of the CMCE, while also examining it from an Indigenous epistemological standpoint, to support the use of this model in a respectful way that is inclusive of Indigenous knowledges (Greenwood et al., 2017). Two-Eyed Seeing, according to Elder Albert Marshall, “refers to learning to see from one eye with the strengths of Indigenous knowledges and ways of knowing, and from the other eye with the strengths of Western knowledges and ways of knowing, and to using both these eyes together, for the benefit of all” (Bartlett et al., 2012, p. 335).

### The Problem of Professional Power

Although the CMCE acknowledges and is concerned with the hierarchical top-down power relations that often accompany professional status and authority, it does not actively challenge the power inequality within the professional–client relationship. It suggests to occupational therapists that “the professional claim of expertise and competence needs to be handled carefully” (Townsend et al., 2007, p. 111). There are attempts within the CMCE to be critical about the unequal power dynamic by highlighting the necessity of power-sharing throughout the model. However, threaded throughout the model there is also a concept of professionalism that upholds hierarchical ordering of abilities and knowledge, derived from a classist system. Within health care, these values and expectations represent mainly the upper-middle class. This is directly contradictory to many Indigenous knowledge principles that value non-hierarchical relationships, emphasize humility and de-emphasize self-importance (Greenwood et al., 2017; Williams & Snively, 2016). Using a model that perpetuates power relations (despite its express intentions) to understand relationships within Indigenous contexts is inadequate.

Martin (2012) notes that when using Two-Eyed Seeing to reconcile Indigenous and Western knowledges, one worldview needs to not dominate or undermine the other. The notion of professionalism, however, is an amorphous Western cultural concept often used to enforce conformity to a *status quo* (Hordichuk et al., 2015; Martimianakis et al., 2009). This inadvertently privileges Western (professional) knowledge over Indigenous knowledge. The CMCE acknowledges power inequities exist in professional–client relationship, yet still frames the role of therapist as that of a professional or expert knowledge holder. Expecting Indigenous Peoples to conform to top-down power relations could undermine forming culturally safe relationships. This type of coercive expectation can have profound implications for the relationships occupational therapists form with the people they work with when gaining informed consent (Boivin & MacLachlan, 2019). The *status* *quo* of Canadian health care is the continuation of colonial, racist ideologies that requires a continuous counter narrative to change the discourse, and ultimately practice (Horrill et al., 2018). Therefore, a model that endorses social change and promotes actively addressing power inequalities, would also need to root its epistemological foundations outside of a professional–client power relationship. This would not mean ignoring core ideas of occupational therapy as a profession, as professionalism is a legal reality of how health care systems operate.

In dominant approaches, health care is defined as a service being provided to patients or clients; this notion of capitalism can hinder recognition of the complex social relations that impact people's ability to access health care services. In contrast, health care can be understood as “both a clinical and social space” (Horrill et al., 2018, p. 3), and social spaces can be defined as “invisible and contested space[s] in which people from different social positions negotiate access to power and resources in their everyday reality” (Tang et al., 2015, p. 710). Adopting a postcolonial lens that views health care as a social relationship between people, rather than a client–professional service, would provide a way to more clearly address complex social, historical, and political factors and begin developing safer health care for Indigenous Peoples, and others experiencing systemic oppressions (Horrill et al., 2018). The term post-colonial, despite being used heavily in the literature, is contested by some. Smith (2018) notes that it provides a sentiment that colonialism is over, and as such, anti-colonial is a more accurate term for the current efforts to challenge colonial ideologies and colonialism in society.

### The Problem of “Enabling” Another

The expression of a professional hierarchical foundation plays out in various ways throughout the CMCE, one being the language and descriptions used to define what occupational therapists do. Enablement skills and enablement done *by* a therapist *to* another person implies a degree of power over them. To enable is “to make someone able to do something, or to make something possible” (Enable, n.d). The broad intent of enablement in the CMCE being to break down barriers so people can participate more fully in everyday occupations would seem to be a positive reason to name occupational enablement as the foundation of what occupational therapists do. However, in an Indigenous context, using language that implies that someone—likely from outside of the Indigenous community they are working in—has power over others, and that the therapist is doing the work to make someone “able” may be viewed as problematic. Indigenous Peoples have continuously had to deal with the paternalistic treatment of colonial institutions deciding what is best for them and their communities, and have demonstrated their resiliency in the face of attempted genocide (Allan & Smylie, 2015). Implying that a health care worker, who is representative of a health care system that has a history of exclusion and violence towards Indigenous Peoples (Allan & Smylie, 2015), shall be responsible for enabling Indigenous Peoples or communities could be seen as replicating this colonial paternalism. Further, this notion of “making someone able” disrespects how resilient Indigenous Peoples and communities have been, devising strategies for resisting the detrimental impacts of colonialism and racism on their health and well-being.

The CMCE descriptions do state that clients contribute to their own enablement and bring their own enablement skills to the interactions. The CMCE image is structured with arrows representing both client and professional contributions to the same 10 enablement skills, seemingly implying that these skills are what both sides bring to interactions. However, based on the descriptions of what the enablement skills mean—this is not the underlying assumption. Outside of a few of the enablement skills describing reciprocal roles for clients to take part in, the enablement skills are meant to describe how health care professionals, like therapists, carry out the work of client enablement. This limited inclusion of imagining client reciprocal engagement in enacting enablement would serve to contribute to the construction of clients as *only* recipients of health care services. Throughout the model, the professional is presented as the expert who acts while the client is acknowledged as having value and enablement skills, but has a more passive role. This approach may be unsuited for Indigenous contexts as it may contribute to occupational therapists focusing on fixing perceived problems rather than creating space for reciprocal exchanges that build trusting connections (White & Beagan, 2020). The CMCE places importance on respecting the contributions and knowledge clients bring to enablement interactions but does not consistently demonstrate this ideal within the enablement skills.

### Adapt, Advocate and Collaborate: The Problem of Colonial Narratives

*Adapt* is one of the enablement skills that is acknowledged as a strategy clients continuously use in adapting their own occupations. The role of the therapist in adapting is to analyze the environmental context occupations occur in to support finding ways to adapt occupations to that context. This understanding of the need to consider people within the context where they exist aligns well with Indigenous epistemologies. Indigenous approaches to science and understanding the world emphasize the need to interpret subjects embedded within contexts, and cannot be properly understood by reducing them to isolated components (Doherty, 2019; Williams & Snively, 2016). To remain relevant in Indigenous settings, when using the skill of adapting to determine just-right challenges for clients, occupational therapists would need to ensure that when breaking down occupations into various components they do not lose sight of the interconnected contexts of the components. Another way to push the skill of adapting occupations toward greater relevance in Indigenous contexts might be to consider not just how to adapt client occupations, but also employ self-reflexivity to explore how to adapt the role of occupational therapy itself. This would center the notion that understanding context is not just about putting others under a microscope but also about understanding the therapist's context as well (Martin, 2012), challenging the idea that the professional is inherently neutral, objective, unbiased and the health care context is devoid of cultural values and assumptions.

Interconnected with this reflexive adaptation would be how the enablement skill of *advocacy* is framed. Advocacy within the CMCE centers using critical perspectives to bring about change to support justice and inclusion. Bringing critical perspectives would be important for practice in Indigenous contexts in order to address the systemic origins of health inequities and how colonialism has worked to eradicate the foundations of community well-being for Indigenous Peoples (Reading & Wien, 2009). For advocacy to be suitable in working with Indigenous Peoples there needs to be an internal interrogation of how occupational therapists bring colonialism and racism into practice. Embedded in social institutions, especially education, is a constructed ignorance to support the continuation of colonial violence and white/settler privilege (Lavallee & Mushquash, 2016). While being critical is important, it also necessary to go a step further to acknowledge that attempting to reform policy and lobby for systemic change may not go far enough when Canada as a nation is founded on the continued oppression of Indigenous Peoples. As Audre Lorde famously stated, “The master's tools will never dismantle the master's house. They may allow us temporarily to beat him at his own game, but they will never enable us to bring about genuine change. And this fact is only threatening to those … who still define the master's house as their only source of support” (1984, p. 112). In other words, the CMCE encourages therapists to engage in advocacy to support justice and inclusion, yet they must draw on the tools, theories, models, and practices of colonial institutions to do this work. Limiting efforts to truly address colonialism and racism to only working within racist, colonial political institutions will make dismantling these institutions to create new ways of practicing occupational therapy without white supremacy difficult to enact (Grenier, 2020).

Grounding what therapists do in a framework of professional-client hierarchy undermines any attempt to break away from and critique power hierarchies. There are elements within the enabling skill *collaborate* that would be important for Indigenous contexts. For example, Two-Eyed Seeing highlights the importance of cultivating genuine respect for different ways of knowing the world that do not dominate over one another (Martin, 2012); so too does the collaborate skill in the CMCE call for a relationship that actively facilitates working together rather than on behalf of clients. Collaboration challenges the hierarchy of professional experts by understanding clients as experts within this relationship. It highlights the need to be attentive to how power is distributed in health care toward the professional, and the need to actively create space where power is shared. A way to strengthen collaboration would be to ground it in Indigenous collaborative approaches such as Two-Eyed Seeing and Ethical Space. Ethical Space is a model for creating mutual trust, respect, equality, and collaboration for respectful interactions of differing ways of knowing (Ermine, 2007). This would provide an outline of how to approach the therapeutic relationship with humility and reflexivity, providing a structured way of honoring Indigenous contributions (Greenwood et al., 2017). The CMCE focuses on power sharing, which centers on the professional granting power to clients. This incorporates the assumption that clients do not have the ability to assert their own demands and power. Indigenous communities have for centuries been finding ways to resist colonization and challenge White supremacy, so the notion of granting clients and communities power continues a narrative of paternalistic dependence on colonial institutions (Allan & Smylie, 2015). Further, this granting of power does nothing to address Indigenous self-determination.

### Coach, Educate and Coordinate: How Language Influences the Approach

*Coach* and *educate* are two of the enabling skills that also demonstrate this paternalistic top-down understanding. Learning from someone and finding new ways to accomplish goals are not necessarily negative things, but a collaborative approach to learning and exchanging knowledge would challenge professional power hierarchies. In a historical context that includes using the institutional education system of Residential Schools to carry out genocide, and a current context of intergenerational trauma stemming from those experiences, it is highly problematic to continue a hierarchical framework of teacher–student (Nelson, 2012). Similarly, coaching implies instructing, guiding, and encouraging, as an experienced practitioner in a field of performance helps others advance develop and advance their skills. Again, it implies hierarchy.

An approach that supports collaboration and attention to power would prioritize reciprocity in knowledge sharing that creates a community relationship rather than rigid professional boundaries (White & Beagan, 2020). Using Indigenous epistemological understandings of learning as being a community endeavor (Cajete, 1993; Williams & Snively, 2016), that has people learning from and with each other within a specific context, would break down assumptions that one-way transmission of knowledge is necessary and the standard within health care interactions. The expectation that someone needs to be a professional or an expert creates pressure on health care providers to prove their value through expertise, which contributes to a culture of not questioning dominant assumptions (Lavallee & Mushquash, 2016). Therefore, in the context of a racist, colonial system, the assumptions that uphold inequities routinely go unchallenged, benefiting the continuation of the *status quo*.

Exchanging the skills of educating and coaching in the CMCE for *knowledge sharing* might encourage a more collaborative approach. Knowledge sharing allows the expectation that professionals listen to what clients have to share and incorporate this into the therapeutic interaction. It suggests that professionals will take in the expertise presented by clients to reframe their ways of thinking and working with clients. A more genuine and active breaking down of the power of a therapist as an expert is possible if learning with/from clients is the normative expectation rather than just a request to respect client expertise.

Another way of imagining how changing the CMCE enablement skills could disrupt the concept of professional experts who lead is shifting the focus of language from *coordinate*—which is defined as “making different things work effectively as a whole” (Coordinate, n.d) to *harmonize*—which is “to successfully combine different activities, systems, and ideas” (Harmonize, n.d). Another option may be to change enablement to facilitation, which delineates more of a partnership with inherent power sharing rather than a client being “enabled” to do something. Shifting focus away from language that assumes professional leadership toward language that implies being a part of a larger whole, part of a reciprocal relationship, allows for a greater possibility for humility and a balanced way of framing health care as a social relationship (Horrill et al., 2018). This type of framework would more closely align with Indigenous worldviews that de-emphasize individual self-importance in favor of community and environmental interdependence (MacLachlan, 2010; Williams & Snively, 2016).

### From Client-Centeredness to a Strengths-Based Framework

A further reimagining of the CMCE to increase its suitability within an Indigenous context might be to embed it in a decolonizing strengths-based framework instead of client-centeredness (Gerlach, 2018; Gibson et al., 2018; Gibson, 2020). The purpose of client-centeredness within the CMCE would seem to be to create a positive and collaborative working relationship between clients and professionals, in which client needs are at the forefront. However, there is clearly an acceptance of a hierarchical client–professional relationship threaded throughout the CMCE. This suggests that the use of client-centered language, while well intentioned, may also be a rhetorical device to advance a professional agenda of proving the value of occupational therapy within dominant health care systems (Hammell, 2013). This can be demonstrated by interrogating the assumptions underlying the way effective client-centered enablement is defined: “Enablement would be effective first and foremost from the client perspective. However, occupational theorists, managers, funders, and policy makers would agree that enablement is based on sound financial management and accountability to promote health, well-being and justice through occupation” (Townsend et al., 2007, p. 130). By implicitly accepting that well-being and justice can and should be measured alongside balancing budgets, the model obscures how dominant political and social priorities are intentionally cultivated to prioritize profit over peoples’ well-being, in neo-liberal systems of health care (Hammell, 2019).

This is especially problematic for Indigenous Peoples who have faced chronic and deliberate underfunding to community infrastructure, housing, education, health care and other supports for social determinants of health (Reading & Wien, 2009). In Canada, this approach to resource management serves the purpose of maintaining settler-colonial power and privilege (Lavallee & Mushquash, 2016). In order for decolonization to occur there needs to be a rejection of ways of knowing that reinscribe colonial agendas (Martin, 2012). This means moving away from defining people as clients whose well-being should be quantified through financial accountability. Occupational therapy cannot be invested in maintaining dominant social, political, and economic contexts that maintain health inequities if social well-being and justice are genuinely the goals of the profession. Either it can maintain domination and colonization or it can work to liberate and decolonize (Anderson-DeCoteau, 2016). There should be no in-between. Continuing to use the “masters tools” (Lorde, 1984) betrays ongoing commitments to reconciliation, and social and occupational justice (CAOT, 2018).

The decolonizing strengths-based framework outlined by Gibson et al. (2018; see also Gibson, 2020) provides an example of how the CMCE could be embedded into the social, political, and historical contexts of colonial society and demand therapists critically reflect on the ways people, professions, and systems reproduce inequalities. Gibson and colleagues’ approach to developing this strengths-based framework is an example of how practice models can be developed with the communities they are meant to serve. This framework also places an emphasis on the necessity of Indigenous Peoples evaluating the extent of decolonization in therapeutic processes. Using the framework's decolonizing critical reflection and evaluation, the CMCE can be transformed into a cycle of preparation, action, and reaction instead of a linear relationship with a beginning and end. Preparation would mean reflecting on the embedded context of the environment, personal history, and position within this context for all participants within the relationship. A social justice and human-rights approach considers social determinants that may make entering the health care relationship unsafe or difficult for particular groups and individuals. A complex understanding of cultural safety would provide an approach to address safety in practice (Greenwood et al., 2017). The critical reflection and evaluation would provide space within the model for analyzing and building on these experiences in preparing for the next therapeutic encounter.

## Conclusion

The work of rising to the TRC Calls to Action rests with settler Canadians as well as Indigenous Peoples, working in partnership. The work of transforming a profession is not easy, or comfortable. As Chontel Gibson has stated:

We all need to do the hard work, which involves critical reflections and changing the systems, structures, and processes. This hard work will result in transforming the way we work together, transforming the way we practice as a profession, and transforming our identities, both personal and professional. (2020, p 18)

That transformative work cannot be done with continued, unquestioned use of the “master's tools.” It is time to critically examine dominant models and theories for their colonial underpinnings.

Summarizing the overall changes proposed here, instead of a CMCE we might have something along the lines of a Social Model of Strengths-Based Occupational Facilitation. Imagining a relationship entrenched in social interconnectedness, that demands critical reflection and action to inform a move toward decolonizing occupational therapy within Indigenous contexts is just beginning the process of interrogating and dismantling dominant Eurocentric theories and practice models. If the profession of occupational therapy aims to address the recommendations of Canada's Truth and Reconciliation Commission (2015), there is a need to critically examine current practice, assessments, theories, and models, including the ways they reproduce a colonial narrative. Notably, we need more Indigenous occupational therapists within the profession to better meet the needs of Indigenous clients; but educating those therapists to employ tools and ways of thinking steeped in colonial racism does them and Indigenous clients a grave injustice.

## Key Messages

• The CMCE is positioned as a client-centered model—however there is a clear hierarchical client–professional relationship threaded throughout.
• OTs need to take meaningful action towards reconciliation by critically examining their own practices as well as the practice models and assessments used and how these reproduce a colonial narrative.
• The gold standard for the OT profession is to create practice models and assessments in collaboration with Indigenous Peoples, communities, and organizations to ensure their relevancy.

## Supplemental Material

sj-jpeg-1-cjo-10.1177_00084174211042960 - Supplemental material for Critiquing the Canadian Model of Client-Centered Enablement (CMCE) for Indigenous Contexts Click here for additional data file. Supplemental material, sj-jpeg-1-cjo-10.1177_00084174211042960 for Critiquing the Canadian Model of Client-Centered Enablement (CMCE) for Indigenous Contexts by Carly Hunter and Tara Pride in Canadian Journal of Occupational Therapy
