# Supplementary figures and images for: Critiquing the Canadian Model of Client-Centered Enablement (CMCE) for Indigenous Contexts
Source: Can J Occup Ther. 2021 Oct 19;88(4):329–39. doi: 10.1177/00084174211042960 (PMC8733346; doi:10.1177/00084174211042960)

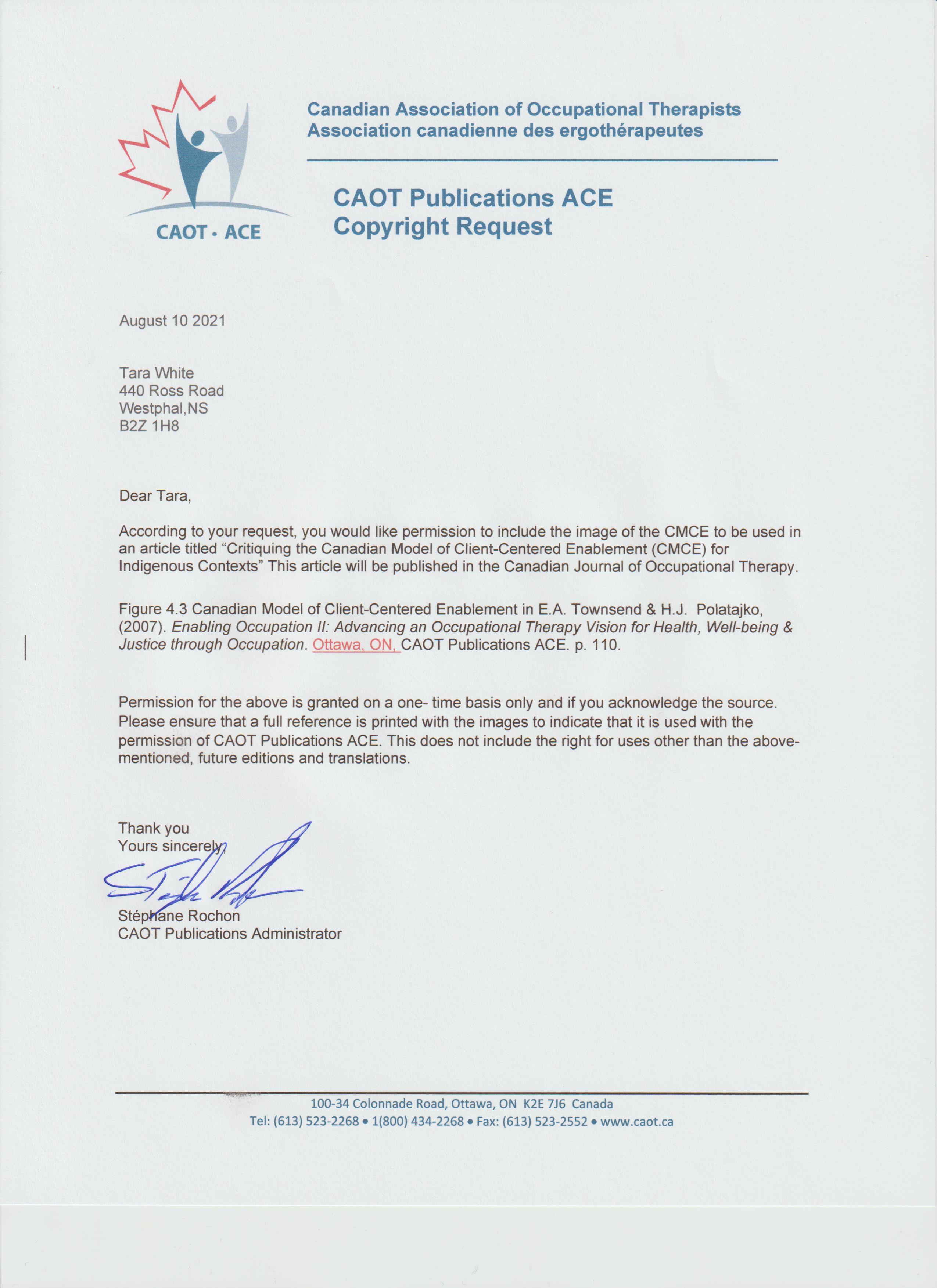

Supplement: sj-jpeg-1-cjo-10.1177_00084174211042960 - Supplemental material for Critiquing the Canadian Model of Client-Centered Enablement (CMCE) for Indigenous Contexts [file sj-jpeg-1-cjo-10.1177_00084174211042960.jpeg]
